# Disambiguating authenticity: Interpretations of value and appeal

**DOI:** 10.1371/journal.pone.0179187

**Published:** 2017-06-26

**Authors:** Kieran O’Connor, Glenn R. Carroll, Balázs Kovács

**Affiliations:** 1 McIntire School of Commerce, University of Virginia, Charlottesville, Virginia, United States of America; 2 Graduate School of Business, Stanford University, Stanford, California, United States of America; 3 Management Group, Yale School of Management, Yale University, New Haven, Connecticut, United States of America; University of Naples Federico II, ITALY

## Abstract

While shaping aesthetic judgment and choice, socially constructed authenticity takes on some very different meanings among observers, consumers, producers and critics. Using a theoretical framework positing four distinct meanings of socially constructed authenticity–*type*, *moral*, *craft*, and *idiosyncratic*–we aim to document empirically the unique appeal of each type. We develop predictions about the relationships between attributed authenticity and corresponding increases in the value ascribed to it through: (1) consumer value ratings, (2) willingness to pay, and (3) behavioral choice. We report empirical analyses from a research program of three multi-method studies using (1) archival data from voluntary consumer evaluations of restaurants in an online review system, (2) a university-based behavioral lab experiment, and (3) an online survey-based experiment. Evidence is consistent across the studies and suggests that perceptions of four distinct subtypes of socially constructed authenticity generate increased appeal and value even after controlling for option quality. Findings suggest additional directions for research on authenticity.

## Introduction

Although social scientists recognize the extent to which authenticity is increasingly valued in organizational contexts [1–3], the interpretations associated with it often differ substantially [4, 5]. That is, some very different meanings of the authenticity concept are salient among individual consumers as well as social scientists [6–8].

Consider, for example, the following items: a tape measure once owned by President Kennedy or a harpsichord played by Johann Sebastian Bach [9, 10], a first edition of *Huckleberry Finn* [11], genuine Chicago Blues music [12], a person’s own true self at work [13], authentic Texas barbecue [14], and comeback rock and roll tours in Europe [15]. Each of these examples has been deemed “authentic” by individuals and scholars, and many have claimed that these examples carry added value because of that general ascription.

One intriguing aspect of these examples is their application of the same conceptual label—authenticity—that carries with it so many distinct meanings. Such a broad use of the conceptual label underscores the importance attached to anything defined by it, while at the same time neglecting the nuance embedded within it as such a rich and varying construct. While impactful, authenticity has become inherently ambiguous and difficult to elucidate. Extant attempts to compare and synthesize the different meanings of authenticity in use do not always clarify matters. As we review below, various typologies of authenticity cut and slice ideas and usage in different ways (see, for example [16–19]), but some still miss major distinctions drawn by other scholars. Others give lists of definitions without much interpretation or theoretical guidance. We find this conceptual disorganization unsatisfying and not very useful for understanding and predicting behavior; it also undermines what we see as key insights about authenticity in the social world.

More strikingly, we know of no systematic quantitative attempt to assess empirically the usages of the various meanings of authenticity. Given the widespread belief in the importance of authenticity as a driver of behavior in advanced contemporary economies, the dearth of evidence addressing this issue is unsettling. Accordingly, in this analysis, we seek to address this gap in research on authenticity. We posit generally that individuals recognize and use the many distinctions in authenticity drawn by philosophers and social scientists. In investigating this claim, we attempt to document the behavioral implications, if any, of various interpretations of authenticity for consumer behavior, including valuation and willingness to pay more for items perceived as authentic.

Our efforts here reflect an attempt to develop empirical evidence about the recognition and use of differing conceptions of socially constructed authenticity in organizations. Using archival analysis and a series of experimental tests, we explore the range of discrete meanings in evaluations and decisions about authenticity among individuals. In doing so, we rely on a broadly inclusive framework from organizational theory that integrates most other contemporary usages of the concept, by positing four kinds of authenticity—*type*, *moral*, *craft*, and *idiosyncratic*—which we detail below. Accordingly, while this study investigates the empirical foundations of a specific theory, it also speaks to broader questions about socially constructed authenticity across a range of organizations and products.

## Theoretical framework

### Variation in meanings of authenticity

Considered generally, authenticity is an attribution that individuals assign to certain objects and activities. *Nominal* or *indexical* authenticity [4, 18] typically relies on knowable facts in reference to an objective assessment of authenticity—such as whether a Roman coin was minted during the rule of Marcus Aurelius. By contrast, the *socially constructed* attributions involve judgments of more abstract value above and beyond the factual and hedonic characteristics of the object [1, 3, 12, 20, 21].

Whereas nominal authenticity is objectively verifiable (although perhaps only with great effort), socially constructed authenticity is often more elusive. Accordingly, objective facts are less important here than interpretations of those facts [22]. For example, a beer might evoke hedonic value through its hoppy aroma and malty flavor, while at the same time it may also convey a more symbolic or abstract sense of authenticity. This occurs, for instance, when a consumer contemplates “the brewer’s goals (say, attempting to produce a nineteenth century German style ale), the ingredients used (organic malt and hops grown in a special region) and the production techniques (small batches made in old refinished equipment and using no chemicals or modern quality controls)” [23]. This interaction between individuals’ subjective interaction with an experience, organization, or object produces and enhances the value of its socially constructed authenticity [24]. Yet, this process elicits diverging interpretations of what is authentic, which can make it impossible to assess definitively, especially because the meanings can evolve over time [12]. The abundance of meanings and their elusive nature may also explain why so few have attempted to assess the range of interpretations and uses of socially constructed authenticity and the unique value generated by each.

Social scientists attempting to reconcile various abstract conceptual meanings associated with authenticity often offer typologies, where they describe and juxtapose the differences. For examples, among the many available others, see: Peterson [5], who lists various definitions from the dictionary; Beverland [6], who gives several different interpretations found among commercially marketed products; and Dutton [4], who describes generic conceptual differences found in common language usages.

A common assumption of many analyses holds that authenticity depends on the interpretation of the observer, critic or consumer. Some view these interpretations as emanating from systematic audience-based differences across groups, audiences and markets as well as across time [5, 12, 25]. The focus of our present empirical analysis is to explore individual behavior across several common contexts for consistent evidence of distinct interpretations. While interpretations may vary within and across these dimensions, one focal aim is to investigate what foundations, if any, express a clear and consistent set of distinct authenticity meanings.

### A typological framework of authenticities

Here, we use an established theoretical framework to understand different interpretations of authenticity and to guide our empirical work (see [23]). While others have previously attempted to develop scales from the ground up to measure different authentic meanings, such as in food [26, 27] and tourism [28], we build on this theoretical framework both because it relies on Weberian ideal types and because it claims (at least implicitly) to be comprehensive in its coverage. The ages old appeal of Weberian ideal types in organizational theory rests in part on audience recognition of the theorized types. A Weberian ideal type is supposed to reflect the key conceptual dimensions of a phenomenon, even though no perfect realization may exist (think of the graphic illustrations in a field guide to birds or plants). Ideal types may overlap or intersect, meaning that an entity might be characterized by more than one type. For Weber [29], a crucial criterion for an ideal type was that it possesses “adequacy of meaning,” implying it is recognizable and interpretable in the social world—audience-based, in other words. Comprehensiveness in a typology implies that it encompasses all the regular usages of the concept. Thus, even if distinct meanings may vary to some degree across time and place, we sought to explore consistency of the underlying foundations that would remain consistent across these shifting patterns.

The framework we use recognizes as distinctive four different and generalized meanings of authenticity [23]. Using the typology of this framework, we define each of the four meanings below: *Type authenticity* describes something that is true to its associated type or genre [4, 30], such as a steakhouse in the U.S. with leather booths, white tablecloths and prime beef, often aging in a visible display. This meaning evokes an institutionalized system of categories, which organizations and their products fit or not [31]. Although this meaning may appear similar to notions of heritage or tradition in other cultural contexts, type authenticity is distinct from these related constructs. Consider, for example, traditions of hazing in athletic and military organizations, which could be regarded as high in tradition and heritage, but low in authenticity. *Moral authenticity*, on the other hand, concerns the sincerity of the (usually moral and values-driven) choices behind an organization and its products [32], such as Tom’s Shoes or Warby Parker glasses. Here, moral authenticity may reference the non-economic logic of decision making to uphold the principles of the organization or its founders and members [33].

Stemming from these two foundational meanings are two evolved expressions of authenticity. *Craft authenticity* suggests a transformation or evolution of type authenticity, where the question is whether something is made using the appropriate techniques and ingredients (an example would be a micro-distillery such as Coppersea in Hudson Valley New York), without necessarily fitting into a specific category. The emphasis here rests more on process than on typicality. The fourth and final meaning, *idiosyncratic authenticity*, emerged out of the ubiquity of claims about moral authenticity; it assigns a commonly recognized historical quirkiness to the organization, product or service (an example would be McSorley’s Old Ale House in Manhattan).

Seeing these four meanings of authenticity as Weberian ideal types implies that it may be difficult to find perfect concrete examples of each and that specific organizations might simultaneously possess more than one type. On the other hand, this ideal-types framework provides the opportunity for empirical tests to be comprehensive in nature. One of the central claims of the literature is that authenticity as an ideal or virtue is widely sought after by individuals in modern society. Whether there are consistent foundations of different meanings across different contexts, however, is an empirical question that few, if any, have tried to test.

How, then, should empirical analysis proceed in studying ideal types of authenticity with a typology? An initial effort needs to involve describing the types and linking them to real entities in compelling ways; that is, this effort requires convincing others that the theorized types contain insights that help us organize the world analytically. For the four types studied here, much work of this kind is available already; it is qualitative in nature and consists of providing examples, cases, and situations that fit one type or another (for reviews, among many others see [5, 12, 23]). The next effort, which we pursue here, likely needs to involve collecting and analyzing systematic or quantitative data pertinent to the types and the typology.

But what exactly should those data demonstrate? One step involves empirical verification that phenomena are perceived consistently with the conceptual claims of the typology. This is similar to what Merton [34] called “establishing the phenomena” except that here we are not simply establishing its social or real existence but also examining its consistency with a theoretical construct. Given Weber’s [29] criterion of “adequacy of meaning,” such proof implies to us that the relevant individuals will recognize and respond to representations about the four specific types of authenticity. And given that the typology purportedly taps into distinctive (if possibly overlapping) elements of authenticity, empirical evidence should also show that there is some discrimination among types.

An important related step, which we focus on here, requires assessing whether individuals who identify and recognize each subtype of authenticity also bestow it with value. That is, do they consider the version of authenticity they see as imbued with value, or is each subtype merely a proxy for one another? Accordingly, we develop testable hypotheses designed to explore the relationship between different authenticity meanings and the unique value associated with each.

### Types of authenticity and valuation

Individuals frequently make assessments of the value of organizations and their offerings. The value of a product or service offered to an individual can be considered as the person’s “overall assessment of the utility of a product [or, brand, service, or experience] based on perceptions of what is received and what is given” [35]. At its core, value is an assessment of what is given up for what is received [36], but the dimensions of value often include emotion, social context, quality, and price [37].

The perceived authenticity of an item or service often depends on the identity of its underlying producer organization. That is, individuals often focus on producer organizations to assess authenticity because it is the organization that makes the items, offers the services, and provides the experiences for consumers. Accordingly, the underlying producer organization’s identity and structural design is typically evaluated when individuals make and verbalize authenticity attributions. For instance, a system of internal production, rather than outsourced production, is often associated with authenticity [38]. It is also, of course, the producer organization that stands to profit from such attributions of authenticity. Moreover, many modern organizations go to great lengths to project an image of authenticity, believing it will create value for them [39].

Perhaps because of the value placed on socially constructed authenticity, organizations sometimes attempt to convey different authenticity types in an effort to manage social perceptions and protect positive organizational images, identities, and reputations [40]. In turn, individuals interpret and use these cues to make choices that feel more meaningful, predictable, and consistent [41, 42]. The end result is a dynamic pattern in which individuals identify and value what they perceive as authentic across a range of organizations and product offerings, something that motivates organizational leaders strategically to make those claims as attractive as possible. A central question is whether audiences recognize and value different meanings of authenticity and whether leaders’ current understanding of organizational identity is sensitive enough to communicate strategically these differences.

Indeed, Kovács and colleagues [20] provide evidence that restaurants perceived as generally authentic receive higher value ratings by consumers than those not regarded as authentic, all other things equal, including especially quality. But their test lumps together all producers that might express any of the four subtypes of the typology. On a broad level, this convergence makes sense because it is possible for organizations to embody any number of combinations of these four types. For example, Alice Waters’s Berkeley restaurant Chez Panisse could be seen as embodying all four types of authenticity, as could Sukiyabashi Jiro, the critically acclaimed Tokyo restaurant celebrated in the documentary *Jiro Dreams of Sushi*.

Organizations embodying any of the four distinct types, or any combination thereof, likely enhance ascriptions of their generic authenticity, as Kovács and colleagues [20] demonstrated. Such research, however, conflates all authenticity meanings by considering all possibilities as the same. The potential analytical problem of this approach is that one type of authenticity could drive all increased value associated with an organization or objects, while other expressed meanings may be present but contribute little to no associated increases in value. In this and other work on the topic, the specific value of the full range of diverse meanings has been overlooked or slighted. By contrast, we aim here to uncover this nuance by demonstrating the link between divergent meanings of authenticity and the unique value associated with each type. Thus, we drill down a notch into the theory and propose that if the ideal types of the typology are valid, then we should see higher value ratings associated with each.

**HYPOTHESIS 1 (H1): *When used by individuals to describe organizations and objects as authentic*, *each of the four meaning types will generate higher value ratings***.

The preceding hypothesis underscores two important components of our theorizing. First, we predict that individuals recognize and express language consistent with each of the four authenticity meanings in an unconstrained context (e.g., open-ended text responses). Second, when recognized and attributed to an organization and its products, each of these meanings is assigned unique added value.

Many analysts believe that authenticity carries a more special meaning for those who see it—entities regarded as authentic are accorded some special significance. Although the nature of this attribution may differ by usage and context, the distinction of “special-ness” does not. As Lindholm [43] observes, “authenticity, in its multiple variations, exalted and ordinary, is taken for granted as an absolute value in contemporary life”. When it pertains to goods and services transacted in a market, the value accorded authenticity is usually regarded as conveying literal meaning. Here, socially constructed authenticity conveys abstract value, which signals a premium above and beyond utilitarian distinctions [44]. Thus, what is considered congruent with each of the four types of authenticity expresses meaning greater than what is perhaps fundamentally practical and functional. Consistent with H1, objects perceived as authentic are perceived as more valuable than other non-authentic objects with the same characteristics. Taken more literally, if authenticity does indeed impute value, then it should not only affect individual valuation as H1 claims, it should affect a buyer’s willingness to pay: authentic objects that embody any of the four types should also elicit a price premium.

**HYPOTHESIS 2 (H2): *Individuals are willing to pay more for products that embody any of the four authenticity meanings***.

## Three studies in a multi-method research program

To explore these hypotheses, we conducted a programmatic series of related studies that complement each other by using different data sources and different methods. Table 1 describes and compares the basic design dimensions of the studies. It is our intention that the studies complement each other in compiling evidence that overcomes the specific objections one may rightfully make of any single study (or its methods) viewed in isolation. To assess the robustness of findings across samples, the studies also vary in the types of authentic items featured, the usual prices of the featured items, the types of individuals studied, the degree of real life involvement by individuals, as well as the observational methods and sample sizes.

**Table 1:** Key design parameters of the three studies.

Study	N	Population sampled	% Female	Mean Age	Item Offered	Method	Quality Confound
Study 1	1,249,426	Restaurant patrons in NY, LA, and Dallas	NA	NA	All restaurants	Archival analysis of consumer reviews	Controlled econometrically
Study 2	96	University population	57.6	20.5	Chocolate	Behavioral lab experiment	Controlled strictly (high)
Study 3	298	U.S. population	40.3	37.2	Burgoo	Online experiment	Choice option

The framework we use was developed within the context of food and dining. So, in conducting tests, we stayed in this domain as well, using online review data of restaurants voluntarily entered by real users in the course of their lives; it is thus likely based on the actual experiences of individuals. Our first study uses data based directly on individual recognition of authenticity types in real world entities rather than materials designed by scientists based on theory. We collected and analyzed these data, relating the attributions of authenticity made by reviewers in their comments to the value ratings they assigned to restaurants. That is, this study measured attributions about the authenticity types made voluntarily without prompts rather than manipulating them, as in subsequent experiments. In this study, we also developed and used several observable measures of restaurant quality as controls.

The two subsequent studies experimentally manipulated the range of authenticity meanings in different controlled environments: (a) in a university-based behavioral laboratory (Study 2) with actual chocolate samples and corresponding manipulations of product descriptions, and (b) in an online experiment (Study 3) where a sample drawn from a broad population viewed and read descriptions of unfamiliar products (burgoo stew) to minimize the influence of past experience. Thus, we aimed to test contexts that featured real products and descriptions that were both known and familiar to participants as well as those that were real, but relatively unknown and uncommon, so as to minimize prior product knowledge and isolate authenticity meanings even further.

A potential confounding factor in assessing an item’s perceived authenticity is its perceived quality. That is, consumers may be using authenticity as a label or signal of underlying quality [6]. Accordingly, it seemed imperative to attempt to control for quality among the options we tested. We did this in three distinct ways in these experiments. In Study 1, we measured the number of times reviewers used words typically associated with quality and then controlled for this variable econometrically. In Study 2, we removed the choice option for quality and instead controlled for it by offering options that were all comparably high quality. In Study 3, we included a fifth high-quality choice; this option lacked any form of explicit authenticity, thereby isolating individuals who had a strong preference for quality. In all the experiments, we also measured and controlled for age, gender, level of income, and level of education.

In accordance with the PLoS One journal standards, we note here that the Stanford University Institutional Review Board (IRB) specifically approved the studies reported in this manuscript. All human subjects whose data are used in this manuscript gave written informed consent for each experimental study by signing a consent form determining that their data would remain anonymous throughout the entirety of the study design and in perpetuity. These written consent procedures were approved by the Stanford University Institutional Review Board.

## Study 1: Archival analysis of online reviewers

### Study design

#### Description

Study 1 explored how individuals identify and use the four types of authenticity in their daily experiences by investigating online restaurant reviews and the value that individuals assign to such attributions. Two separate components comprise the study. The first component recruited participants to an online wiki survey (the platform known as All Our Ideas) and attempted to identify the words they used to describe each type of authenticity and to assign weights to each word. The second and core component of the study used an archival dataset of online reviews of dining consumers compiled by [20]. Consumers voluntarily decided if and when to review a restaurant with the online system. Reviews include a rating for the restaurant as well as qualitative textual comments. We analyzed both. This study allowed us to examine the relationship between attributions of any of the four types of authenticity and user value ratings in a real-world setting.

#### Participants

The study used two datasets generated by consumers: the initial online survey to generate authenticity keywords to use in analyzing textual comments, and a second archival dataset used to explore H1. We recruited the survey participants (N = 182) from a national online panel managed by a large research university in the United States. Participants completed the survey for a small monetary remuneration. The archival dataset contains 1,271,796 reviews written by 252,359 unique reviewers of 18,869 restaurants by consumers in metropolitan New York City, Dallas, and Los Angeles from October 2004 to October 2011.

#### Method

Two components comprise the study, a first identifying the authenticity attributions for each of the four types and a second evaluating impact of each type on consumer reviews and value ratings. (We discuss the more technical first component in S1 Appendix. We report relevant data and more detailed analyses from it in S2 Appendix and S3 Appendix.) Participants provided written consent prior to completing the first study, which were approved by the university’s institutional review board and confirmed that their data would remain anonymous. To summarize, in this component we extended the framework by Kovács and colleagues [20] to identify attributions that individuals make about each of the four types of authenticity. Individuals viewed a series of word pairs and selected which word within each pair best represented a specified authenticity type. The results of this component are numeric scales for words used to refer to each of the four authenticity types. We initially developed one scale for each authenticity type. However, to assess the robustness of the findings, we subsequently devised three ways to calculate each scale. We refer to this set of measures as *ATS1*, *ATS2* and *ATS3* (referring the Authenticity Types Scale #1, #2 and #3); we used them to assess and score the texts of each online review.

The focal data of Study 1 come from the second component. These data came from reviews of restaurants in three metropolitan areas–Los Angeles (LA), Dallas, and New York (NYC)–posted to a dining-oriented consumer-driven review website (known as Yelp.com). The website was founded in 2004 and generates its reviews through a voluntary process in which any patron can go online and write a review. Each review captures four pieces of information linked to the restaurant that we used: (1) a reviewer identification code; (2) a user value rating of stars, ranging from one to five as an integer; (3) a text review of unlimited length; and (4) the date of the review. Reviewers can enter a rating and the text of a review in whichever order they want; the website does not force any sequential ordering. The data analyzed cover the entire period of a restaurant’s entry in Yelp.com, the longest of which could start in October 2004, and end at the time of the data collection in October 2011. In total, 1,271,796 reviews were provided across the three cities.

### Variables, measurement and estimation

#### Value ratings

Measured as the number of stars (out of five) assigned to the restaurant by the reviewer, this variable is the outcome of main interest in this study (Hypothesis 1). The distribution of ratings in the data shows a tendency toward the higher scores: across the three cities, roughly 25% received a score of five, about 40% received a 4, approximately 20% a 3, around 10% a 2 and 5% a 1.

#### Authenticity

We content analyzed [45] the text of each review entered for each restaurant. Our goal was to assess to what degree the reviewer described the restaurant as authentic or inauthentic along each of the various dimensions based on the four types. The keywords and corresponding scores in each of the authenticity scales described in S1 Appendix were used to assign type-specific authenticity scores to each review. That is, for each review we averaged the authenticity values of all the words that appeared in the review and were also listed in the relevant authenticity keyword list. A notable and highly attractive feature of this measurement strategy is its complete passivity and unobtrusiveness. There was no priming or prompting or stimulus designed to elicit a consumer’s view of the authenticity of a place. Rather, we relied entirely on unsolicited voluntary comments regarding authenticity.

Using the four authenticity scales constructed with the wiki surveys from the first component of the study, we used content analysis to analyze the text of each review recorded in the sample, aiming to assess the extent to which the reviewer described the restaurant as authentic or inauthentic. To do so, we created a computer script that went through the text of all 1,271,796 reviews. In each review, the script identified the set of authenticity-related keywords, and using the authenticity scales in S3 Appendix, it calculated the sum of the authenticity scores for each review. The computer script also accounted for the negative usage of the authenticity terms. For example, it coded ‘not authentic’ as ‘inauthentic.’ Thus, we calculated for each review twelve authenticity values: four using ATS1, four using the ATS2, and four using the ATS3.

#### Control variables

Following Kovács and colleagues [20], to rule out alternative explanations we controlled for various organizational and reviewer-level attributes. We wanted to estimate the effects of authenticity above and beyond quality. We attempted to do so in two different ways. First, we analyzed the text of each review and parsed out quality-related words in order to develop word counts of these words. Second, we obtained data with separate ratings on the quality of food from the independent Zagat Restaurant Guide. We used the 2011 Zagat Guides of Los Angeles, New York City, and Dallas for these ratings. We matched these ratings on a restaurant-by-restaurant basis to the review dataset from Yelp. Because Zagat reviews only a fraction of all restaurants, regression models using this control result in a significantly reduced sample size.

Additional controls used in the statistical analysis include (1) ownership structure (family-owned/chain/other); (2) niche width (number of distinct cuisines the restaurant is categorized in); (3) price (as measured in four categories in Yelp.com–“cheap,” “moderate,” “spendy,” and “splurge”); (4) the age of the restaurant at the time of the review; (5) the number of restaurants in the same cuisine in the same city as well as for the average rating of those similarly categorized restaurants; (6) the extent to which reviewers were actively engaged in the online reviewing community (i.e., their “domain enthusiasm”); (7) the geographical location of the restaurant, using zip code dummies, and (8) the year, using dummy variables.

#### Level of analysis and estimation

Because we think that this level is most appropriate for the questions examined here, we analyzed the reviews at the review-level. Value ratings are made by individuals, and thus these judgments represent individual perceptions of authenticity. For ease of interpretability, we present estimates of linear regression here. We note that the findings presented here remain consistent (on substantive issues) in alternative modeling frameworks such as ordered logit regressions and regressions conducted at the organizational level.

### Findings

Study 1 investigated Hypothesis 1, the claim that perceived authenticity of any type of a restaurant leads to higher value ratings. Table 2 shows estimates of models of user value ratings. Models 1 and 2 use *ATS1*, models 3 and 4 use *ATS2*, and models 5 and 6 use *ATS3*. S1 Appendix shows additional models that estimate the effect of type, moral, craft, and idiosyncratic authenticity separately.

**Table 2:** Regression estimates of user value ratings on authenticity types.

	Authenticity Types Scale #1 (*ATS1*)		Authenticity Types Scale #2 *(ATS2*)		Authenticity Types Scale #3 *(ATS3*)	
	(1)	(2)	(3)	(4)	(5)	(6)
Type authenticity	0.021***	0.021*	0.088***	0.055***	0.110***	0.091***
	(0.007)	(0.012)	(0.004)	(0.006)	(0.004)	(0.006)
Craft authenticity	0.617***	0.627***	0.281***	0.290***	0.591***	0.561***
	(0.008)	(0.012)	(0.004)	(0.006)	(0.005)	(0.007)
Moral authenticity	-0.460***	-0.547***	0.022***	0.082***	0.169***	0.202***
	(0.005)	(0.007)	(0.007)	(0.012)	(0.010)	(0.016)
Idiosyncratic authenticity	0.036***	0.109***	0.065***	0.083***	0.086***	0.097***
	(0.005)	(0.007)	(0.004)	(0.006)	(0.004)	(0.006)
No. of words in the review	-0.002***	-0.002***	-0.002***	-0.001***	-0.002***	-0.001***
	(0.000)	(0.000)	(0.000)	(0.000)	(0.000)	(0.000)
Price	0.002	-0.020***	-0.000	-0.025***	-0.001	-0.026***
	(0.001)	(0.003)	(0.001)	(0.003)	(0.001)	(0.003)
No. of reviews for restaurant	0.184***	0.064***	0.182***	0.065***	0.182***	0.065***
(in thousands)	(0.003)	(0.004)	(0.003)	(0.004)	(0.003)	(0.004)
Age (years)	-0.011***	0.001	-0.014***	-0.001	-0.014***	-0.001
	(0.001)	(0.001)	(0.001)	(0.001)	(0.001)	(0.001)
Mean rating for cuisine in city	0.740***	0.533***	0.761***	0.548***	0.760***	0.549***
	(0.007)	(0.011)	(0.007)	(0.011)	(0.007)	(0.011)
Domain enthusiasm of reviewer	-0.031***	-0.015***	-0.033***	-0.016***	-0.033***	-0.016***
	(0.001)	(0.001)	(0.001)	(0.001)	(0.001)	(0.001)
No. restaurants in city with same cuisine	-0.007***	-0.018***	-0.003***	-0.014***	-0.004***	-0.015***
(in thousands)	(0.001)	(0.001)	(0.001)	(0.001)	(0.001)	(0.001)
Family-owned	0.114***	0.003	0.122***	0.009	0.123***	0.010
	(0.008)	(0.015)	(0.008)	(0.015)	(0.008)	(0.015)
Chain	-0.009***	0.000	-0.009***	0.000	-0.009***	0.000
	(0.000)	(0.001)	(0.000)	(0.001)	(0.000)	(0.001)
Niche width	-0.001	0.002	0.001	0.004	-0.000	0.003
	(0.002)	(0.003)	(0.002)	(0.003)	(0.002)	(0.003)
High-quality keywords	0.145***		0.154***		0.154***	
	(0.001)		(0.001)		(0.001)	
Low-quality keywords	-0.536***		-0.549***		-0.549***	
	(0.002)		(0.002)		(0.002)	
Zagat’s food rating		0.072***		0.075***		0.075***
		(0.001)		(0.001)		(0.001)
Constant	1.268***	0.762***	1.187***	0.650***	1.194***	0.650***
	(0.034)	(0.051)	(0.034)	(0.052)	(0.034)	(0.052)
Zip code dummies included	Yes	Yes	Yes	Yes	Yes	Yes
Year dummies included	Yes	Yes	Yes	Yes	Yes	Yes
Observations	1,249,426	528,190	1,249,400	528,190	1,249,400	528,190
R-squared	0.179	0.115	0.170	0.101	0.170	0.099
Log-likelihood	-1.817e+06	-771225	-1.824e+06	-775693	-1.824e+06	-775693

*Note*: Dependent variable: User value ratings. Standard errors are reported in parentheses.

*** p<0.01

** p<0.05

* p<0.1

When the scales are entered into the regression equation individually, the estimates show that each of the four types of authenticity contributes significantly to the consumer value rating (see S1 Appendix). This pattern clearly supports Hypothesis 1. In models 1 and 2, the joint effects of the four subtypes are estimated when entered together in the same equation. While the models reveal positive effects for type-, craft-, and idiosyncratic authenticity, moral authenticity is associated with a negative coefficient. However, this model is not well-specified: the high Variance Inflation Factor (VIF = 12.81) indicates collinearity ([46] recommends 10 as a cut-off value). High correlations among the values of the untreated *ATS1* likely generated this situation. So, in models 3 to 6, we present alternative regression estimates of the joint effects of the authenticity subtypes using *ATS2* and *ATS3*, which exhibit less multicollinearity. Consistent with H1, these estimates show clearly that each authenticity type contributes uniquely to user value ratings, even after controlling for restaurant quality.

Comparing the magnitudes of the estimated authenticity coefficients in Table 2 is informative. The mean user value rating in the sample is 3.67 with a standard deviation of 1.14. So, a coefficient in the .5-.6 range indicates that a restaurant perceived as authentic (e.g., craft authenticity = 1) on average receives a consumer value rating one standard deviation higher than a restaurant that is perceived as inauthentic (e.g., craft authenticity = -1). Note also that the four authenticity types differ in the strength of their contributions to consumer value ratings. While the exact importance of the four authenticity types differs across specifications, the better specified combined models (models 3–6) show a consistent pattern: craft and moral authenticity exert the strongest effects. The other two authenticity types, type and idiosyncratic, are weaker, albeit still positive and statistically significant.

### Study 1 discussion

Distinguishing the different meanings associated with the various authenticity subtypes is a first step in assessing the unique value associated with each. The first component of Study 1 explored these various meanings by categorizing the natural language used by real consumers to describe their experiences in real organizational settings, establishing an empirical basis for adequacy of meaning among the four distinct subtypes of authenticity. Next, we explored whether each subtype was predictive of corresponding increases in value ratings, while controlling for the other three subtypes and for producer quality, among other factors. This first test demonstrates the recognition and value associated with each type. Nonetheless, there are possible limitations of these archival data, including survival and self-reporting biases. Although the issue of fictitious reviews may be a concern for any archival online review data, the website we use (Yelp) actively deploys a proprietary filtering algorithm that flags suspicious reviews. Although it is not public, this filtering system removes what some scholars estimate to be approximately 16% of reviews in order to limit false reporting and strengthen review reliability [47]. Additionally, all real-world settings in these data include organizations that can express any of the four authenticity meanings, or any combination thereof. To address these concerns, we designed the next two studies to isolate each of the subtypes experimentally, using language similar to the keywords categorized in Study 1 to test the relationship between each subtype and increased value.

## Study 2: Chocolate tasting, willingness to pay, and choice

### Study design

In the next two studies, we relied on experimental psychological methods commonly used in similar multi-study designs (see [48, 49]). Organizations can strategically express any of the four meanings, leading buyers who recognize any combination of these types simply to infer greater general authenticity and greater appeal. The aim of Study 2 was to explore experimentally the unique appeal and value generated by each of the four types in a controlled context in which we could parse the framework’s four meanings into four corresponding archetypal organizations: producers focusing predominantly on type, craft, moral, and idiosyncratic authenticity. While the benefits of Study 1 demonstrate external validity of the archival dataset, in the next two studies we attempt to focus more on the four distinct meanings by isolating each one from the other, something impossible to do with other confounds in real-world field contexts such as Study 1. Still, we aimed to maintain a compelling realistic setting by testing our hypotheses in the context of actual chocolate samples.

In this study, participants ate four pieces of chocolate in a series of taste tests. We led them to believe that each sample came from one of four high-quality producers and that we were interested in their taste preferences. In reality, each sample was identical and from the same package of chocolate. In this design, the only difference across taste-tests was the treatment information that we provided to manipulate experimentally each authenticity subtype. By holding constant the taste stimuli, we hoped to better understand the role of our treatment (manipulated authenticity) on evaluations and willingness to pay for the sample associated with each meaning. We held constant product quality by instructing participants that all chocolate samples came from comparably high-quality producers. After reading about and tasting each chocolate, we instructed participants to evaluate each sample by reporting their willingness to pay compared to a category anchor price consistent with the in-store price per small package of the actual chocolate used in the study.

#### Method and participants

Ninety-six individuals (57.6% female; *M*_age_ = 20.53, *SD*_*age*_ = 2.43) from a university population participated in the experiment for a nominal fee. Participants provided written consent prior to completing the study, consistent with the consent procedure that was approved when the institutional review board approved the study, and this procedure stipulated that their data would remain anonymous after the study. Participants arrived at a research lab arranged to showcase a long table holding several glass bowls, each of which contained small samples of chocolates, serving tongs, and tasting trays. An experimenter presented each participant with a packet of four cards, each of which contained a description of one ostensibly legitimate chocolate producer written to signal one of the four authenticity types. Participants were told that the study intentionally standardized the visual presentation of these materials so that the identity of each producer would not be revealed to them or influence their judgments (this helped to explain the blacked-out producer names and uniform font printed on the cards used as our experimental stimuli; see S4 Appendix).

Participants were instructed to draw a card at random and to read the producer information before tasting the corresponding chocolate sample related to that card. After reading the card and tasting the chocolate, participants then rated the chocolate sample on all measures (see below). To capture participants’ true preferences among the four types of authenticity meanings and to address incentive compatibility, participants were given accurate information that they would receive a full bar of their most-preferred chocolate at the end of the study. There were no restrictions on available choices among the four types and no limit to the number of bars per type, so participants were unconstrained to reveal their true preferences both to rate each chocolate and to select their preferred chocolate at the end of the study.

#### Assessment and measures

After tasting, participants responded to four queries on seven-point Likert scales indicating the extent to which they found each chocolate to be *enjoyable*, *appealing*, *delicious*, and *disgusting*, the last of which was reverse-coded (*1 = Strongly Disagree*, *7 = Strongly Agree*; alphas for all items within each authenticity type > .87), and reported the price they would be willing to pay for this particular chocolate relative to the one-dollar category price. Next, participants drew another card at random from among the remaining three and responded to the same set of questions after tasting the corresponding chocolate, and so on until they had read about and tasted all four of the chocolate samples. Because the experimental procedure involved deception, we took great caution to complete post-experimental funneled debriefing [50], which revealed no recognition of the deception or hypotheses. In the final step of the experiment, after tasting all four identical samples, participants recorded their preferred option and received a small sample package to take home.

### Findings

We sought to explore whether participants would differentiate among the four types of authenticity in their evaluations of each sample. Again, quality was held constant in the experimental manipulation, all chocolate samples were identical, and the order of authenticity meanings was random. First, an omnibus test comparing all products against the category norm revealed an 87% price premium on average for all four authenticity types combined, *t*(95) = 6.230, *p* < .001. Individually, participants were willing to pay more for products representing each of the four subtypes of authenticity: Type = 88%, *t*(95) = 5.91, *p* < .001; Moral = 94%, *t*(95) = 6.47, *p* < .001; Craft = 76%, *t*(95) = 5.42, *p* < .001; and Idiosyncratic = 93%, *t*(95) = 5.32, *p* < .001.

**Fig 1:**
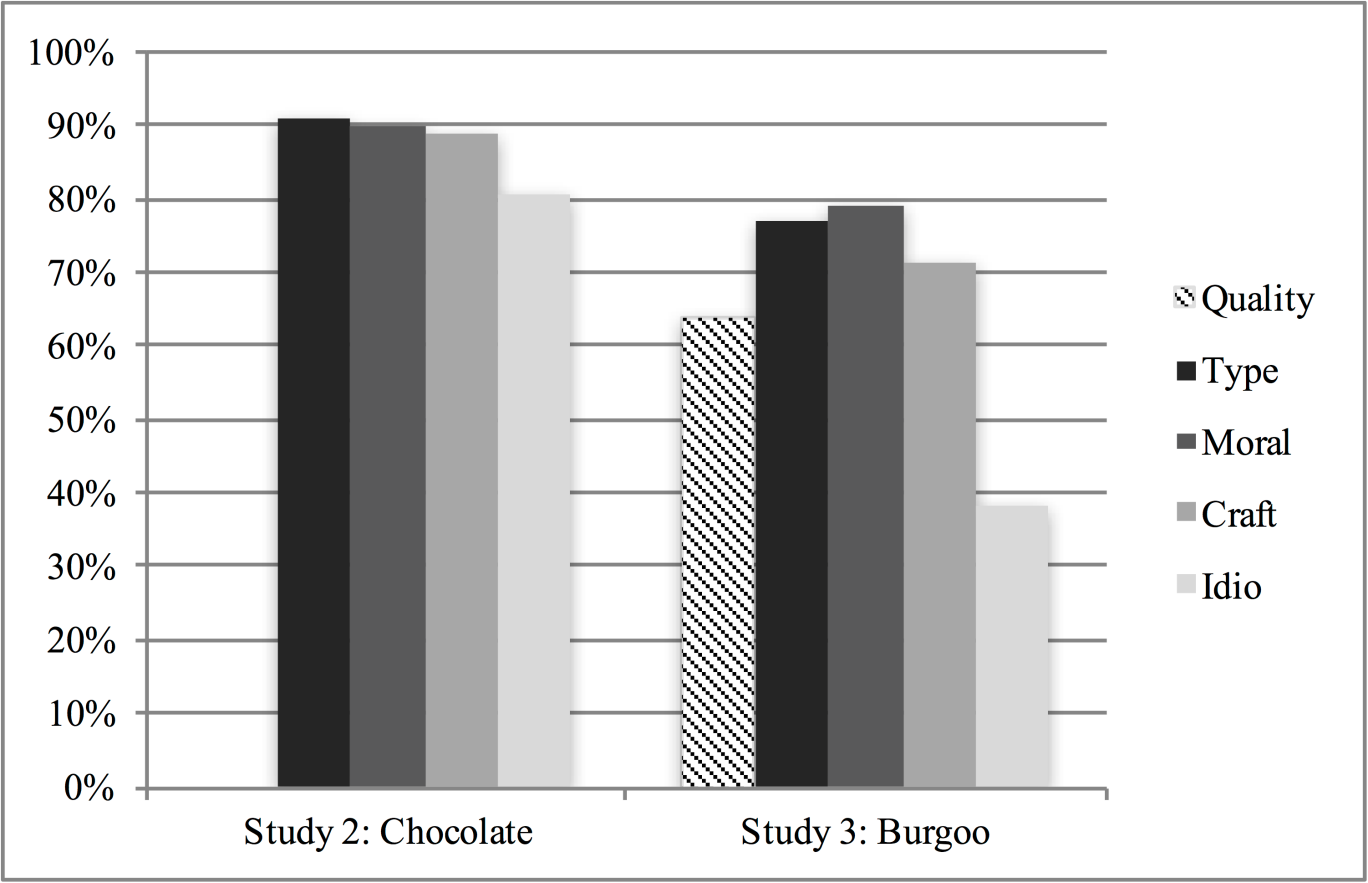
Percentage of individuals willing to pay more than the category price by authenticity subtype choice, studies 2 & 3.

The willingness to pay premium was not driven by bi-modal distributions split by those willing to pay more and others willing to pay the average. Most participants (77%) expressed a willingness to pay more for each authenticity subtype (see Fig 1). If we look specifically at the amount that individuals were willing to pay for their own choice (reported before their choice preference at the end of the study), the reported premium was even stronger compared to the non-selected subtypes combined: Type, *M* = $2.37 vs. $1.73; Moral, *M* = $2.18 vs. $1.83; Craft, *M* = $2.56 vs. $1.57; Idiosyncratic, *M* = $2.22 vs. $1.82.

To explore further the relationships between our experimental treatments, producer appeal, and value, we averaged the scores for appeal, enjoyment, deliciousness, and disgust (reverse-coded) to create a composite *appeal* scale for each chocolate. Despite the uniformity of all physical chocolate samples, participants did differentiate among them based on the manipulation materials expressing each authenticity subtype. The reported appeal of each chocolate, controlling for the reported appeal of all other chocolates, predicted WTP for that respective option in a series of tests, *B*_*Type*_ = .600, *t*(95) = 3.335, *p* = .001; *B*_*Moral*_ = .502, *t*(95) = 3.190, *p* = .002; *B*_*Craft*_ = .471, *t*(95) = 3.349, *p* = .001; *B*_*Idio*_ = .940, *t*(95) = 14.864, *p* < .001. For example, rating the *type*-authentic chocolate as more appealing was associated with a higher willingness to pay for that chocolate, controlling for the appeal of each other chocolate. First, this suggests that individuals tasting the same sample in four repeated iterations differentiated their perceptions of each. We argue that these differences in perceptions were driven by the treatment materials that manipulated each authenticity sub-type in isolation. Second, these differential perceptions lead to different levels of reported appeal, which in turn were tied to an increased ascription of value measured by willingness to pay.

Running a similar set of analyses predicting behavioral product choice shows that appeal for each type also drives preferences for that option (dummy-coded: 0 = options not-chosen, 1 = chosen option): *B*_*Type*_ = .266, *t*(95) = 4.991, *p* < .001; *B*_*Moral*_ = .293, *t*(95) = 5.953, *p* < .001; *B*_*Craft*_ = .213, *t*(95) = 5.187, *p* < .001; *B*_*Idio*_ = .307, *t*(95) = 6.264, *p* < .001. Again, rating the *type*-authentic chocolate as more appealing predicted actual behavioral choice for that chocolate at the end of the study.

### Study 2 discussion

In Study 2, we intentionally held constant the stimulus in a choice menu by using only one type of chocolate across four sequential taste tests. This allowed us (a) to manipulate with controlled language each authenticity subtype in isolation, and (b) to explore different perceptions and valuations of the exact same stimulus as judgments co-varied with each authenticity meaning expressed. In other words, the chocolate samples used were all identical, and the only differences across the four taste tests were the experimentally manipulated materials describing each producer as an exemplar for each subtype. These manipulations, which participants read before tasting each chocolate, seemed to affect their judgments of the product tasted, including its gustatory appeal and their valuation of it. Perhaps surprisingly, no participants expressed suspicion that the chocolate samples were identical during a funneled debriefing procedure as part of the standard debriefing procedure [50]. Because some deception was used in this study, we acknowledge the ongoing discussion about its merits in the general discussion below and point readers to relevant materials to consider if readers plan to utilize a similar methodology.

While confirmatory and consistent with Study 1 results, these findings nonetheless may be influenced, in part, by the design of the study. As a preliminary study, we intentionally held quality constant in the manipulated materials (e.g., “all chocolates are equally high-quality”). We also held constant the reference price point, consistent with the in-store price of a small package. Given these features, it is possible that participants would report a higher willingness to pay for any option they preferred and selected, compared to both the dollar-value reference point and all other options tasted. We acknowledge this possible limitation of the study design and attempted to address it in Study 3 by including a fifth option unrelated to the subtypes, representing a purely high-quality, non-authentic option, which we describe in detail below.

## Study 3: Choice and willingness to pay

### Study design

#### Method and participants

To address possible alternative explanations of findings from Study 2, we conducted an online survey-based experiment in which participants reviewed ostensibly real but fictitious descriptions of a consumable item in a randomly ordered sequence and responded to a series of questions about them. As in Study 2, we manipulated the organization and product descriptions in the experimental materials that were designed to highlight the relevant signifiers for each of the four types of authenticity, consistent with the keywords described in Study 1. In this study, we also included a fifth option to exemplify quality (see S4 Appendix for detailed item descriptions). After reading each of the five descriptions (four authentic, one quality), participants evaluated each option on several dimensions, including the price they would be willing to pay and their preference for the most appealing option. Such hypothetical forced choices have been used across a wide range of settings and have demonstrated high degrees of within and out of sample predictive power for actual choices [51–53]. To ensure that our manipulations evoked the specific types of authenticity intended, we also conducted a series of manipulation checks for each item description with an independent sample to avoid any demand effects in the present study (N = 149; Mean age = 35.0; 68.8% female). Participants in the focal sample ranged in age from 18 to 68 (*Mean* = 37.6). Similar online samples have been shown to be at least as representative of the U.S. population as traditional subject pools with regard to gender, race, age, and education [54], and more representative than in-person convenience samples in contemporary social science research [55]. As with Study 2, all participants provided written consent approved by the university’s institutional review board prior to completing the study, which confirmed that all participant would remain anonymous during and after the study.

Individuals first read a scenario about buying a real but highly unfamiliar inexpensive food item: burgoo soup. We deliberately selected this product as a more controlled test of the four authenticity sub-types. In Studies 1 and 2, participants experienced products that are popular and relatively well known (restaurant experiences, chocolate), and as such, they may have brought to bear prior expectations and knowledge about the specific study context and experiences beyond their perceptions of authentic meanings. Here, we aimed to further isolate each of the four types by using a real context, but one that is essentially unknown except in a small specific locale. Burgoo is a unique regional style of meat stew common in Owensboro, Kentucky and local rural environs, often using mutton and a variety of woodland meat cooked for long periods of time. The text of the scenarios read as follows:

*“You are going to a nearby grocery store to buy a stew that is named ‘burgoo.’ When you get to the store, you ask the clerk where the burgoo is*. *The clerk takes you to an aisle where you find the following different kinds of burgoo, all priced roughly the same. On the next page you will read about 5 different types of burgoo stew, and decide which one you would like to buy to share with some friends for dinner.”*

Participants viewed five descriptions of producer organizations, one for each authenticity meaning plus one signifying high quality with no specific mention to any meaning of authenticity. To minimize any possible social desirability response bias, participants signed up for the study with an anonymous online user ID and remained anonymous throughout the study. It was known to all participants that there would be no channel for the experimenters to contact participants during or after the study and that no identifying information would be linked to their data afterward. After viewing all options, participants recorded their preferred option and expressed their willingness to pay for it. The additional fifth quality-only option in this design allows us to address a possible alternative explanation in Study 2. With the quality-only option, it is possible to assess whether individuals are simply willing to report a higher willingness to pay for any preferred option, or whether individuals assign higher value to different meanings of authenticity compared to the purely high-quality option that does not evoke authenticity.

### Findings

As an important first step in this design, as shown in Table 3, participants typically recognized that each authentic object exemplified the type of authenticity it represented among the stimulus choice set. For example, the morally authentic burgoo was perceived as the most moral among the choice set. Consistent with previous studies, these data confirm that individuals recognize and distinguish among the various types of authenticity based on parsimonious and controlled (manipulated) descriptions of different products.

**Table 3:** Responses to manipulation checks of the four authenticity types and percentage of individuals recognizing each of the four authenticity types accurately, pre-test.

	Attribute Query:				
Item Description	Quality	Type	Moral	Craft	Idiosyncratic
**Quality manipulation**	**5.66 (1.14)**	4.90 (1.09)	5.30 (1.11)	4.99 (1.25)	4.48 (1.16)
	**66.4%**	27.5%	45.6%	30.9%	26.8%
**Type manipulation**	5.10 (1.11)	**5.50 (1.15)**	4.94 (1.22)	5.52 (1.17)	4.59 (1.11)
	32.2%	**62.4%**	38.3%	42.3%	30.9%
**Moral manipulation**	5.30 (1.29)	4.38 (1.41)	**5.48 (1.42)**	4.83 (1.38)	4.43 (1.12)
	55.0%	25.5%	**65.8%**	23.5%	45.6%
**Craft manipulation**	5.30 (1.13)	5.14 (1.33)	4.81 (1.41)	**5.66 (1.19)**	4.72 (1.01)
	40.9%	42.3%	32.2%	**51.0%**	31.5%
**Idio. Manipulation**	4.88 (1.20)	4.77 (1.42)	4.70 (1.26)	5.55 (1.31)	**5.17 (1.14)**
	30.9%	31.5%	30.2%	49.7%	**52.3%**

*Note*: In these manipulation checks, we asked participants to rate each of the five types of burgoo (or handbags) along each of the dimensions of importance: type, craft, moral, idiosyncrasy, and quality (e.g., *How moral is X burgoo*? and *How idiosyncratic is X burgoo*?). Entries in the table show the percentage of participants who rated each item highest or equally high on its respective attribute compared to other products rated on the same attribute (e.g., rating the moral item as high or higher on the moral attribute than any other item on the moral attribute). Thus, the table represents *vertical* comparisons. Dominance by the diagonal show the manipulations worked as intended.

Participants systematically preferred the authentic options compared to the quality option (58.7% vs. 41.3%), *X*^2^ = 9.07, *p* = .003, *df* = 1. This pattern was supported by their willingness to pay more for their preferred choice, consistent with H2, *M* = $4.54, *SD* = 1.32, *t*(296) = 7.025, *p* < .001. Despite the possibly obvious alternative explanation that WTP could be due to confirmation bias or post-choice rationalization [56–59], individuals who selected authentic options were still willing to pay a significantly higher premium compared to individuals who selected the quality option, *M*_*authentic*_ = $4.68, *SD* = 1.34, *M*_*quality*_ = $4.33, *SD* = 1.27) *F*(1, 296) = 5.07, *p* = .025. This pattern was also true for each authenticity type (*M*_*Type*_ = $4.41, *SD* = 1.55; *M*_*Moral*_ = $4.81, *SD* = 1.30; *M*_*Craft*_ = $4.61, *SD* = 1.16) with the exception of Idiosyncratic (*M* = $4.29, *SD* = 1.57). Together, this pattern confirmed Study 1 findings, which showed the strongest effects of moral and craft meanings, with weaker effects for the other two types, especially here for idiosyncratic authenticity, which we discuss more below. In supplementary analyses (available upon request), we found no significant differences across age, level of income, and level of education.

### Study 3 discussion

Using a parsimonious design, Study 3 aimed to explore perceptions and evaluations of a highly unfamiliar class of producers (e.g., burgoo soup makers) who each exemplified a distinct authenticity meaning. To address a potential alternative explanation raised by Study 2, we also included a fifth quality-only option that we designed to be equally attractive in terms of quality, but not evocative of any authenticity meanings. Consistent with these aims, participants recognized and distinguished among the different options according to our intended manipulations of each authenticity meaning. Further, participants here also distinguished among the five options and preferred any authenticity meaning over an equally high-quality option. Last, while participants reported a price premium regardless of their preferred option–even if it were the high-quality alternative–they were willing to pay more for most authenticity subtypes compared to the quality option.

## General discussion

Our efforts here constitute a first major step in establishing the empirical existence and importance of various widely discussed interpretations about authenticity. In a general sense, the study represents a kind of existence proof attempting to demonstrate that four ideal types possess “adequacy of meaning” for consumers. The research program relied on multiple diverse methods to explore several types of objects and producer organizations, documenting a unique set of empirical studies about this topic.

First, we used archival data from an online user review system to test whether patrons recognized and valued each of the four types of authenticity in their assessments of real organizations. Individuals’ own language conveyed distinct interpretations of authenticity, each of which produced significant increases in unique value among organizations embodying them. Next, we examined responses to two controlled experiments, one behavioral and one online, using item descriptions and words similar to keywords uncovered in Study 1 to highlight and experimentally parse each of the various hypothesized types of authenticity.

This analysis represents the first broad set of empirical tests exploring the unique value added among a comprehensive set of four distinct meanings of authenticity. In general, we find consistent evidence that individuals: (1) express willingness to pay more for choices they make about authentic items; (2) find authenticity appealing and assign it higher value even when controlling for quality; and (3) recognize and distinguish systematically among the four types of authenticity. We regard these findings as supportive of the hypotheses.

To date, prior studies have conflated the various authenticity meanings by focusing on a few conceptual differences or by lumping together any of the many possible interpretations expressed by organizations. This work often superimposed scholars’ own construed definitions of authenticity to categorize organizations, objects, and experiences. Other work tests only a limited range of authenticity interpretations, and some leave the construct ambiguous by looking at any possible interpretation as valid. We view this general situation as potentially frustrating and confusing; accordingly, we have attempted here to demonstrate the importance of studying distinct meanings empirically and the value added by each.

Our work here is preliminary in the sense that it is the first of its kind to systematically document the use and unique value of each meaning. At the same time, we think such preliminary steps mark a major contribution to the growing literature on authenticity. Our studies here make at least two new contributions. First, we initiate empirical testing about the importance of specific ideal-types of authenticity long acknowledged by the collective body of work on the topic, but not yet tested systematically. Second, instead of imposing our own interpretation to determine the kinds of authenticity present in organizations, we use the data of individuals’ own language and usage expressed in archival data (Study 1) and test individuals’ own recognition of possible ideal types in Studies 2 and 3. We believe these findings mark an important contribution to this increasingly important characteristic among organizations and consumers and will spark further study to examine related questions raised by this work.

One notable contribution of these studies is the implications it raises for managers and organizations alike. If audiences recognize and value these different types of authenticity, they imply that producers could theoretically adopt strategies based on distinct meanings that align with and emphasize core organizational components most strongly. Patterns of findings here suggest this as an important question that managers and organizational leaders should not overlook. Nonetheless, recent work has also documented the risks that producers face if they actively assert authenticity claims themselves, exposing them to a new liability [60].

### Limitations and future directions

Despite these contributions, it is important to note several limitations of the work that we hope will encourage more empirical tests in this area. First, we determined a-priori that the focus of these studies would be an existence of proof that consumers recognize, distinguish, and value each of the four authenticity meanings in our theoretical framework. It is still an important question whether consumers and other individuals consistently prioritize among these four types. While we think it is important to develop hypotheses about such a ranked order, our view was that the first and higher order of business should be to build an adequate foundation for such theorizing, which is what we tried to do here. A related and equally important question is what specific cultural, contextual, and other factors might moderate the ordered preferences among these four types. Here, too, our aim was first to establish recognition, distinction, and valuation for the core underlying meanings among these types across several different contexts, price points, product types, and levels of familiarity. These settings, though, were not intended to be exhaustive. We regard the findings reported here to be encouraging for the existence of such a framework in the real word, but the extent to which these vary by culture, status, and other moderating factors is beyond the scope of this analysis. These questions animate a broader research agenda that we intend to pursue and hope others will as well.

One related and specific question raised by the present studies is the relatively lower evaluation of idiosyncratic authenticity. As others have theorized, this may stem from the fact that it is perhaps the rarest of the four subtypes [23] and possibly more difficult for organizations to capture because it involves elements of history and provenance that would be difficult, if not impossible, to manufacture. Alternatively, even if its existence is more widespread than some suspect, it is possible that idiosyncratic authenticity is valued less by audiences, perhaps because its value is contained in one-time experiences, thereby making it less appealing over time. This preference could produce lower scores on some empirical measures of value that explore prospective experiences with such products and organizations. Last, it is possible that idiosyncratic authenticity will produce weak main effects because audiences ascribe value to it only after other conditions (e.g., quality or other subtypes) have been satisfied. If this were true, future studies could explore different combinations of subtypes that might reveal a more dynamic and interactive value of idiosyncratic authenticity.

Additionally, we assert that an important contribution of these findings comes from the collection of studies in a multi-method design. Admittedly, any given study trades off some limitations in order to emphasize another important contribution. We did our best to address these limitations with the design of each study in the collection. For example, the Study 1 archival data, collected from more than one million real diner reviews, emphasize a unique demonstration of our tests with high external validity, while trading off the possibility that each authenticity meaning may have co-occurred in any restaurant setting. We addressed this directly, first by developing the list of keywords in a concurrent study that allowed us to econometrically control for other subtypes. We also addressed this by crafting materials in Studies 2 and 3 that isolated each subtype experimentally. In Study 2, we could use these stimuli in a realistic setting in which participants consumed four sequential tastes of the same real chocolate sample, while believing each was distinct and embodied a unique authenticity meaning. This design, however, traded some external validity for a more internally valid experimental setting. It also would have been impossible to maintain internal validity while also testing a perfectly representative sample. As a result, we collected data for Study 3 to address some of these concerns with participants drawn from samples known to be more representative than traditional subject pools like that used in Study 2, and with a design that maintained participant anonymity to address the possibility of a social desirability response bias in the in-person Study 2 lab context. To address these concerns, in turn, Study 3 features a more hypothetical design that trades off external for internal validity. However, the strength of Study 2 –with real taste experiences in a unique controlled design–addresses any hypothetical bias problem or possible risk of incentive incompatibility. Taken together, the consistency of findings and reliability of results presents a compelling case for the hypotheses tested in settings.

To create some of these realistic experiences, experimental deception was used to manipulate each set of authenticity materials. We would like to emphasize the care that needs to be taken when using deception experimentally and to follow the guidelines for these procedures outlined by university ethics review boards, as we did in these studies. For completeness, it is also worth noting the different attitudes regarding the use of deception in experiments across disciplines. While the practice is commonly used in psychology, sociology, and their related fields [61], it is proscribed primarily among experimental economists [62] for at least three pragmatic reasons related to “pollution” of participant pools as a public good: (1) that participants deceived in one study may alter behavior in subsequent studies after losing trust in experimenters and experiments, (2) that common knowledge of deception may cause participants to alter behavior in subsequent studies even if they do not have direct experience being deceived, and (3) that even if deception is not used in some studies (e.g., economic participant pools) its use in other disciplines (e.g., psychology and sociology) may cause spillover effects influencing all experimental work [63, 64]. For these reasons and others, the use of deception in experiments may be seen as normative and accepted in some peer-reviewed publications [61], while it is summarily rejected in others, including several leading economic journals [62, 65]. Nonetheless, even among economists there is no plurality as to which deceptive practices are permissible [65]. Some economists assert that there is little evidence to support forbidding experimental deception [66], and others provide empirical evidence that deception may not produce the kinds of spillover effects feared by many [63]. Last, new perspectives shed light on other forms of deception (e.g., implicit deception, surprise restarts) that are more common in experimental economics, but not yet considered problematic [67]. Obviously, care should be taken when deciding on the appropriate methodological tools for a given study, and analysts considering the use of deception should recognize the complexity of this debate and the impact it might have on a manuscript’s potential for publication in some disciplines.

Last, the present set of studies were tested within U.S. contexts with quasi-representative samples. Despite this, we think the appeal of authenticity reaches well beyond its borders and may be one of the most ubiquitous ideals in the social sciences. We have attempted to cover some of this range throughout the discussion, but acknowledge that future work should continue to test the robustness of our findings across other cultures, regions, and countries. We ourselves are intrigued by the possibility of important new findings in these contexts, especially if and when representative samples in these settings are available.

## Conclusion

Taken together, these studies collectively document a highly consistent pattern of results. Across a wide range of settings, types of products, normative prices, and more, results demonstrate for the first time empirically that participants recognize, distinguish, and value each of four distinct authenticity meanings.

## Supporting information

S1 Appendix Identifying authenticity keywords using all our ideas surveys. (DOCX)

S2 Appendix Summary of AOI keyword surveys. (DOCX)

S3 Appendix Scores of keywords from AOI surveys. (DOCX)

S4 Appendix Product descriptions, study 2 & 3. (DOCX)

S1 Data Study 1 data. (DOCX)

S2 Data Study 2 data. (SAV)

S3 Data Study 3 data. (SAV)
