# Supplementary material for: Disambiguating authenticity: Interpretations of value and appeal
Source: PLoS One. 2017 Jun 26;12(6):e0179187. doi: 10.1371/journal.pone.0179187 (PMC5484484; doi:10.1371/journal.pone.0179187)
Supplement: S1 Appendix — (DOCX) [file pone.0179187.s001.docx]

**S1 Appendix. Identifying Authenticity Keywords Using WikiSurveys**

In this component of Study 1, we extended [20]’s framework to identify attributions about each of the four types of authenticity. The approach respects the consumer’s own interpretations of texts and language, by taking respondents’ comments at face value. That is, we do not infer a broader reference to, say, a specific type of setting as implying authenticity, instead we use the actual words that consumers employ to articulate expressly the authenticity idea or some very close synonym. This conservative approach to measurement holds value in its transparency and ease of replication. We also think this approach helps in understanding public discourse about authenticity.

Implementing this approach required that we first conduct surveys to generate authenticity scales for each of the four types of authenticity. These scales were constructed using separate samples and wiki surveys. In these surveys, participants were presented with a simple instruction, for example, to “please choose the word below that would best describe a restaurant as *morally* *authentic*” and a series of two-word pairs. For each pair of words, the participant was asked to choose which of the two words was the preferred descriptor. Participants could also choose an “I can’t decide” option, in which case they could answer: “because both are good answers” or “neither are good answers.” The pairs of keywords were randomly selected from a keyword list that we describe below. This format was used because it allows for simple and direct comparisons between two words, which places a much lighter cognitive burden on participants than alternative structures such as rank-ordering a long list of keywords in decreasing order of relevance. Each participant was asked to complete at least 50 such comparisons in order to receive the gift card. Each participant answered only one of the surveys, which was assigned randomly to the participant.

The list of keywords used in the surveys came from two sources. First, we generated an initial list of keywords by seeking synonyms of “authentic” and “inauthentic” found in Roget’s Thesaurus and Merriam-Webster’s Thesaurus. This resulted in 56 keywords. Second, participants were allowed to propose new keywords that they considered related to the prompt to which they were responding. Keywords were presented in random pairs, and every keyword could appear multiple times. The resulting authenticity scales can be found in Appendix C.

Not surprisingly, the resulting four authenticity scales are positively correlated. For example, the correlation of the scores along “craft” and “moral” authenticity dimensions is .88, while the correlation between “moral” and “idiosyncratic” authenticity values is .69. These high correlations, on the one hand, are good news as they indicate that the four subtypes of authenticity are related to each other, pointing to the meaningfulness of the concept of authenticity. On the other hand, the high correlations make it challenging to assess the effects of the four authenticity subtypes separately, as including them in a single regression presents estimation problems from multicollinearity.

We find correspondence between our type-specific measures and the general authenticity scores of [20] in that pairwise correlations range from 0.58 to 0.97. Data analysis shows that this correlation mostly results from the lower end of the authenticity scales (i.e., those words most strongly associated with *inauthenticity*). The correlations drop to the 0.23-0.46 range for the subsample of words that are assigned 50 points or more in the general authenticity scale whereas the correlations remain high for the lower end of the authenticity distribution. This indicates that some words with very negative authenticity connotation (such as “fake,” “phony,” and “scam”) score low on all the surveys.

To explore the interrelationships of the four types of authenticity, we conducted a Principle Component Analysis (PCA) on the scores of the keywords. The PCA revealed that a general authenticity dimension explains 85% of the variance in keyword scores. This dimension is a linear combination of the scores along the general authenticity score and the scores along the four types, with, interestingly, approximately the same weights (general authenticity score’s weight: 0.45; type: 0.46; craft: 0.47; moral: 0.45; idiosyncratic: 0.41). These loadings indicate two important facts: first, there exists a concept of authenticity to which the four types contribute; second, the four types form distinct dimensions of authenticity such that the scores along each of the four subtypes cannot be explained with scores along the other dimensions. While correlated, the four scales do differ in certain key words, and the differences in the scores of these words along the four dimensions helped us identify what is unique about each of the authenticity types. We attempted to identify the words that are highly diagnostic for one or few dimensions. For example, the word “offbeat” has a high score on the idiosyncratic authenticity scale, but moderate scores on the other three scales. Words such as “offbeat” helped us separate the four dimensions, while words such as “genuine” or “sham” are less diagnostic as they have similar scores along the four dimensions.

The various word scores could be transformed in many ways to create more distinctive scales for the four authenticity types. For the sake of robustness, here we present two ways of modifying the scales, and later in component 2 of the study show how the original values and these transformed scales help explain how authenticity affects restaurant ratings.

First, we recoded the 0 to 100 scores to a -1 to 1 scale, such that the original 0 score corresponded to -1 (fully inauthentic), the original 50 to 0 (unrelated to authenticity), and the 100 to 1 (authentic). This recoding does not change the correlations, it simply aids in interpreting the scales. We refer to this set of measures as *ATS1* (referring the Authenticity Types Scale #1)*.*

Data inspection shows that there is greater difference between the four types for the keywords that score high on the scales, but there is more overlap in the scores for words that are more authenticity-neutral, the middle ranges. To capitalize on this fact, we created an alternative scale for each authenticity type such that we only included keywords scoring 80 or higher on a dimension. For example, this method included the keywords “genuine” or “real” for the type authenticity dimension. We also included keywords that scored 20 or less on a dimension. For example, keywords “sham” or “unreal.” We refer to this set of measures as *ATS2.* The correlation among these scales is lower, ranging between .61 and .71.

Finally, in a third set of alternative constructs, we let words that are uniquely high or low in specific authenticity types take on a higher weight in those specific scales. To achieve this, we transformed *ATS2* in that we inversely weighted the value of each keyword on each authenticity type with the total number of scales on which the word appears. For example, “genuine” appears in three dimensions. So, on this transformed scale it takes a value of 1/3 on the dimensions where it is included (type, craft, and moral authenticity). “Creative” only appears on the craft authenticity scale, so it is assigned the value of “1”. This procedure ensures that words unique to a specific or only a few authenticity dimensions carry a higher weight. We refer to this set of measures as *ATS3.* The pairwise correlations between these scales range between .05 and .60.

**Table S1: Regression Estimates of User Value Ratings on Authenticity Types**

|  | **Authenticity Types Scale #1 (*ATS1*)** | | | | | | **Authenticity Types Scale #2 *(ATS2*)** | | **Authenticity Types Scale #3 *(ATS3*)** | |
| --- | --- | --- | --- | --- | --- | --- | --- | --- | --- | --- |
|  | (1) | (2) | (3) | (4) | (5) | (6) | (7) | (8) | (9) | (10) |
| Type authenticity |  | 0.435*** |  |  |  | 0.057*** | 0.088*** | 0.055*** | 0.110*** | 0.091*** |
|  |  | (0.002) |  |  |  | (0.008) | (0.004) | (0.006) | (0.004) | (0.006) |
| Craft authenticity |  |  | 0.438*** |  |  | 0.663*** | 0.281*** | 0.290*** | 0.591*** | 0.561*** |
|  |  |  | (0.002) |  |  | (0.008) | (0.004) | (0.006) | (0.005) | (0.007) |
| Moral authenticity |  |  |  | 0.345*** |  | -0.553*** | 0.022*** | 0.082*** | 0.169*** | 0.202*** |
|  |  |  |  | (0.003) |  | (0.005) | (0.007) | (0.012) | (0.010) | (0.016) |
| Idiosyncratic authenticity |  |  |  |  | 0.520*** | 0.123*** | 0.065*** | 0.083*** | 0.086*** | 0.097*** |
|  |  |  |  |  | (0.003) | (0.005) | (0.004) | (0.006) | (0.004) | (0.006) |
| No. of words in the review |  | -0.002*** | -0.002*** | -0.001*** | -0.001*** | -0.002*** | -0.002*** | -0.001*** | -0.002*** | -0.001*** |
|  |  | (0.000) | (0.000) | (0.000) | (0.000) | (0.000) | (0.000) | (0.000) | (0.000) | (0.000) |
| Price | 0.025*** | 0.036*** | 0.034*** | 0.039*** | 0.035*** | 0.034*** | -0.000 | -0.025*** | -0.001 | -0.026*** |
|  | (0.002) | (0.002) | (0.002) | (0.002) | (0.002) | (0.002) | (0.001) | (0.003) | (0.001) | (0.003) |
| No. of reviews for restaurant | 0.197*** | 0.214*** | 0.211*** | 0.215*** | 0.206*** | 0.200*** | 0.182*** | 0.065*** | 0.182*** | 0.065*** |
| (in thousands) | (0.003) | (0.003) | (0.003) | (0.003) | (0.003) | (0.003) | (0.003) | (0.004) | (0.003) | (0.004) |
| Age (years) | -0.015*** | -0.016*** | -0.014*** | -0.017*** | -0.015*** | -0.014*** | -0.014*** | -0.001 | -0.014*** | -0.001 |
|  | (0.001) | (0.001) | (0.001) | (0.001) | (0.001) | (0.001) | (0.001) | (0.001) | (0.001) | (0.001) |
| Mean rating for cuisine in city | 0.910*** | 0.838*** | 0.830*** | 0.878*** | 0.851*** | 0.819*** | 0.761*** | 0.548*** | 0.760*** | 0.549*** |
|  | (0.007) | (0.007) | (0.007) | (0.007) | (0.007) | (0.007) | (0.007) | (0.011) | (0.007) | (0.011) |
| Domain enthusiasm of reviewer | -0.042*** | -0.032*** | -0.031*** | -0.033*** | -0.031*** | -0.029*** | -0.033*** | -0.016*** | -0.033*** | -0.016*** |
|  | (0.001) | (0.001) | (0.001) | (0.001) | (0.001) | (0.001) | (0.001) | (0.001) | (0.001) | (0.001) |
| No. restaurants in city with same cuisine | -0.003*** | -0.009*** | -0.010*** | -0.008*** | -0.008*** | -0.008*** | -0.003*** | -0.014*** | -0.004*** | -0.015*** |
| (in thousands) | (0.001) | (0.001) | (0.001) | (0.001) | (0.001) | (0.001) | (0.001) | (0.001) | (0.001) | (0.001) |
| Family-owned | 0.152*** | 0.131*** | 0.127*** | 0.140*** | 0.136*** | 0.128*** | 0.122*** | 0.009 | 0.123*** | 0.010 |
|  | (0.009) | (0.008) | (0.008) | (0.009) | (0.008) | (0.008) | (0.008) | (0.015) | (0.008) | (0.015) |
| Chain | -0.011*** | -0.011*** | -0.011*** | -0.011*** | -0.011*** | -0.011*** | -0.009*** | 0.000 | -0.009*** | 0.000 |
|  | (0.000) | (0.000) | (0.000) | (0.000) | (0.000) | (0.000) | (0.000) | (0.001) | (0.000) | (0.001) |
| Niche width | -0.005*** | -0.003 | -0.003** | -0.005*** | -0.004** | -0.003* | 0.001 | 0.004 | -0.000 | 0.003 |
|  | (0.002) | (0.002) | (0.002) | (0.002) | (0.002) | (0.002) | (0.002) | (0.003) | (0.002) | (0.003) |
| High-quality keywords |  |  |  |  |  |  | 0.154*** |  | 0.154*** |  |
|  |  |  |  |  |  |  | (0.001) |  | (0.001) |  |
| Low-quality keywords |  |  |  |  |  |  | -0.549*** |  | -0.549*** |  |
|  |  |  |  |  |  |  | (0.002) |  | (0.002) |  |
| Zagat’s food rating |  |  |  |  |  |  |  | 0.075*** |  | 0.075*** |
|  |  |  |  |  |  |  |  | (0.001) |  | (0.001) |
| Constant | 0.687*** | 0.936*** | 0.974*** | 0.817*** | 0.911*** | 1.000*** | 1.187*** | 0.650*** | 1.194*** | 0.650*** |
|  | (0.037) | (0.036) | (0.036) | (0.036) | (0.036) | (0.036) | (0.034) | (0.052) | (0.034) | (0.052) |
| Zip code dummies included | Yes | Yes | Yes | Yes | Yes | Yes | Yes | Yes | Yes | Yes |
| Year dummies included | Yes | Yes | Yes | Yes | Yes | Yes | Yes | Yes | Yes | Yes |
| Observations | 1,249,400 | 1,249,400 | 1,249,400 | 1,249,400 | 1,249,400 | 1,249,400 | 1,249,400 | 528,190 | 1,249,400 | 528,190 |
| R-squared | 0.036 | 0.080 | 0.083 | 0.058 | 0.072 | 0.093 | 0.170 | 0.101 | 0.170 | 0.099 |
| Log-likelihood | -1.917e+06 | -1.888e+06 | -1.886e+06 | -1.903e+06 | -1.893e+06 | -1.879e+06 | -1.824e+06 | -775693 | -1.824e+06 | -775693 |

Note: Standard errors in parentheses, *** p<0.01, ** p<0.05, * p<0.1
