# Supplementary material for: Disambiguating authenticity: Interpretations of value and appeal
Source: PLoS One. 2017 Jun 26;12(6):e0179187. doi: 10.1371/journal.pone.0179187 (PMC5484484; doi:10.1371/journal.pone.0179187)
Supplement: S2 Appendix — (DOCX) [file pone.0179187.s002.docx]

**S2 Appendix. Summary of All Our Ideas Keyword Surveys**

| **Authenticity type** | **Question Asked** | **Number of individuals completing the survey** | **Number of pairwise keyword comparisons** |
| --- | --- | --- | --- |
| Type | *Restaurants are often referred to by type, such as “Chinese restaurant.” Please choose the word below that, in your opinion, best describes a restaurant that is an authentic rendering of its type.* | 43 | 3,249 |
| Craft | *Restaurants often employ chefs and other workers with particular knowledge and skills about buying, cooking and preparing food for diners. Please choose the word below that, in your opinion, best describes a restaurant with authentic craftsmanship.* | 48 | 3,475 |
| Moral | *Restaurants often source, prepare and serve food that reflects certain moral values, such as sustainability or humane treatment of animals. Please choose the word below that, in your opinion, best describes a restaurant embodying authentic moral choices.* | 39 | 2,862 |
| Idiosyncratic | *Restaurants are often known for quirky features of their past, including events occurring in them or things their owners or patrons have done. Please choose the word below that, in your opinion, describes a restaurant which is authentically idiosyncratic.* | 52 | 2,964 |
| Total |  | 182 | 12,550 |
