# Supplementary material for: Disambiguating authenticity: Interpretations of value and appeal
Source: PLoS One. 2017 Jun 26;12(6):e0179187. doi: 10.1371/journal.pone.0179187 (PMC5484484; doi:10.1371/journal.pone.0179187)
Supplement: S3 Appendix — (DOCX) [file pone.0179187.s003.docx]

**S3 Appendix. Scores of Keywords from AOI Surveys**

| **Type authenticity** |  | **Craft authenticity** |  | **Moral authenticity** |  | **Idiosyncratic authenticity** |  |
| --- | --- | --- | --- | --- | --- | --- | --- |
| delicious | 83 | creative | 84 | caring | 90 | unique | 92 |
| genuine | 83 | genuine | 83 | moral | **85** | quirky | 87 |
| real | 83 | skillful | 83 | inspiring | 82 | interesting | 86 |
| skilled | 81 | delicious | 82 | pure | 82 | idiosyncratic | 80 |
| authentic | 79 | authentic | 77 | genuine | 80 | authentic | 78 |
| professional | 79 | expert | 77 | ethical | 78 | creative | 76 |
| honest | 77 | fresh | 77 | truthful | 78 | offbeat | 76 |
| interesting | 75 | professional | 77 | professional | 77 | genuine | 74 |
| skillful | 75 | ambitious | 76 | skilled | 77 | iconic | 73 |
| special | 73 | artful | 75 | decent | 76 | original | 73 |
| creative | 72 | interesting | 75 | heartful | 76 | peculiar | 71 |
| unique | 72 | heartful | 74 | wholesome | 76 | sincere | 71 |
| artisan | 71 | master chef | 73 | authentic | 74 | real | 70 |
| fresh | 71 | real | 73 | creative | 74 | special | 70 |
| old-fashioned | 71 | new | 72 | interesting | 73 | atypical | 69 |
| expert | 70 | unique | 72 | unique | 73 | inspiring | 69 |
| sincere | 70 | inspiring | 71 | faithful | 72 | artisan | 68 |
|  |  |  |  |  |  |  |  |
| *For space consideration, keywords with values between 40 and 70 are excluded from this table. The full appendix is available at request.* | | | | | | | |
|  |  |  |  |  |  |  |  |
| extroverted | 37 | offbeat | 35 | unmistakable | 34 | untraditional | 36 |
| outlandish | 37 | artificial | 34 | unorthodox | 34 | pretentious | 34 |
| orthodox | 36 | extroverted | 32 | orthodox | 33 | usual | 34 |
| quirky | 36 | peculiar | 32 | peculiar | 33 | moral | 33 |
| imitation | 33 | unassuming | 31 | typical | 33 | normal | 33 |
| offbeat | 32 | outlandish | 30 | artificial | 32 | orthodox | 32 |
| unconventional | 31 | unorthodox | 28 | untraditional | 31 | replica | 32 |
| feigned | 29 | deceptive | 24 | extroverted | 29 | assumed | 30 |
| deceptive | 25 | imitation | 23 | imitation | 29 | inauthentic | 25 |
| unorthodox | 25 | unreal | 22 | phoney | 25 | quack | 24 |
| fake | 21 | ersatz | 21 | unauthentic | 23 | artificial | 23 |
| humbug | 21 | impostor | 19 | unreal | 22 | cheat | 23 |
| phony | 21 | inauthentic | 18 | cheat | 21 | imitation | 23 |
| unauthentic | 21 | unauthentic | 18 | deceptive | 19 | phony | 21 |
| cheat | 20 | cheat | 17 | ersatz | 18 | fake | 20 |
| bogus | 19 | untraditional | 17 | impostor | 18 | feigned | 18 |
| phoney | 19 | phony | 16 | phony | 18 | phoney | 18 |
| untraditional | 19 | feigned | 15 | feigned | 17 | unreal | 18 |
| false | 19 | phoney | 15 | dishonest | 16 | deceptive | 17 |
| ersatz | 18 | bogus | 14 | quack | 16 | bogus | 16 |
| faked | 17 | quack | 12 | humbug | 15 | hoax | 16 |
| inauthentic | 17 | faked | 11 | inauthentic | 15 | humbug | 16 |
| scam | 16 | hoax | 11 | bogus | 14 | unauthentic | 16 |
| forgery | 15 | forgery | 10 | faked | 13 | forgery | 14 |
| unreal | 15 | dishonest | 9 | sham | 13 | impostor | 13 |
| impostor | 14 | scam | 9 | forgery | 12 | scam | 13 |
| hoax | 13 | fake | 8 | scam | 12 | false | 12 |
| quack | 13 | false | 8 | hoax | 11 | faked | 8 |
| sham | 11 | sham | 7 | false | 8 | sham | 5 |
| dishonest | 8 | humbug | 6 | fake | 6 | dishonest | 2 |
