# Supplementary material for: Disambiguating authenticity: Interpretations of value and appeal
Source: PLoS One. 2017 Jun 26;12(6):e0179187. doi: 10.1371/journal.pone.0179187 (PMC5484484; doi:10.1371/journal.pone.0179187)
Supplement: S4 Appendix — (DOCX) [file pone.0179187.s004.docx]

**S4 Appendix. Product Descriptions, Study 2 & 3**

| Authenticity Meaning | Study | Product Description | Product Description |
| --- | --- | --- | --- |
| Type | S2 | Chocolate | More than 70 years ago in a small town in the Swiss Alps, a master chocolate maker embarked on a path that would make him one of the world’s greatest chocolatiers. Benito Grazzioni set out on his own to establish the finest cocoa recipes in the word. Now lead by Benito’s great-grandson Lucca, Poco Dolce still produces some of the world’s most quintessential fine chocolates in the same traditional family recipes passed down for three generations. Poco Dolce is the mark of distinguished chocolate. |
|  | S3 | Burgoo Stew | The Bottoms family settled in Western Kentucky and their renowned family stew came to be seen as a quintessential burgoo. Will’s burgoo is still made according to the original recipe of Will Bottoms’ multigenerational Kentucky family, using beef, pork, mutton, game, small wildlife. |
| Moral | S2 | Chocolate | Dolce's artisan chocolates feature only the finest all-natural ingredients- from organic local ingredients and sea salt. All chocolates are produced from cacao beans that are sourced from 100% sustainable farms throughout Central and South America that are part of the Rainforest Alliance Program committed to provide a living wage and safe, healthy working conditions for all producers. We also source local ingredients from farmers markets throughout the area to create seasonal specialties, and produce everything in our fully solar operated facility |
|  | S3 | Burgoo Stew | Bill’s Burgoo uses only organic meats and poultry obtained from animals raised in humane free range conditions. Grown biodynamically, the vegetables, spices and herbs used in its preparation come from local artisanal farms. The burgoo is cooked at local community events organized to raise funds for local farmers in financial distress. |
| Craft | S2 | Chocolate | Hand made in micro batches in our San Francisco production kitchen, DDDDolce's artisan chocolates are produced by our professionally-trained master chocolatiers who have spent years honing their craft with the top chocolatiers across Europe. We start by hand sourcing the finest ingredients, sorting within each lot, simmering our small batches in precise temperature-controlled copper kettles, and cooling immediately on French ceramic racks to capture the essence of each piece. Visit our small production line, by appointment only. |
|  | S3 | Burgoo Stew | Jack’s Burgoo uses only finely butchered meats, including Western Kentucky game birds, jackrabbit, opossum, squirrel, and venison. Seasoned with bouquets of herbs picked fresh from Kentucky woodlands and fields, expert chefs simmer the burgoo for days in large 19th century cast iron pots over an open wood fire. |
| Idiosyncratic | S2 | Chocolate | Our motto “poco pazzo, poco dolce,” says it all: a little creative and a little sweet. While producing some of the finest chocolates, we do so with an air of creativity. To honor the Aztec’s who introduced the world to chocolate, all Brandna are produced in synchrony with the Aztec calendar, using ratios of ingredients in each recipe according to its calculus. To celebrate the year we began, we produce 92 bars per batch—no more, no less. |
|  | S3 | Burgoo Stew | Daniel’s Burgoo is made only on full moon nights in pots rinsed the night before with local Owensboro moonshine. In addition to the usual variety of meats, the recipe also calls for a two dozen cleaned and declawed squirrels’ feet, demands that the burgoo be cooked on a fire built from oak wood collected from at least two separate Kentucky counties. |
| Quality | S3 | Burgoo Stew | John’s Burgoo uses only the finest quality meats, vegetables and herbs. The beef and pork are USDA prime; the poultry is air-chilled; the game comes from certified wildlife habitat farms. The fresh vegetables and herbs are grown in local small farms. |
