# Supplementary material for: Disambiguating authenticity: Interpretations of value and appeal
Source: PLoS One. 2017 Jun 26;12(6):e0179187. doi: 10.1371/journal.pone.0179187 (PMC5484484; doi:10.1371/journal.pone.0179187)
Supplement: S1 Data — (DOCX) [file pone.0179187.s005.docx]

**Study 1 Yelp Data:**

https://www.dropbox.com/s/0rla5w0054sbi7o/PLOS_One_dataforStudy1.zip?dl=0
